# Polarization twist in perovskite ferrielectrics

**DOI:** 10.1038/srep32216

**Published:** 2016-09-02

**Authors:** Yuuki Kitanaka, Kiyotaka Hirano, Motohiro Ogino, Yuji Noguchi, Masaru Miyayama, Chikako Moriyoshi, Yoshihiro Kuroiwa

**Affiliations:** 1 Department of Applied Chemistry, School of Engineering, The University of Tokyo, 7-3-1 Hongo, Bunkyo-ku, Tokyo 113-8656, Japan; 2 Graduate School of Science, Hiroshima University, 1-3-1 Kagamiyama, Higashihiroshima, Hiroshima 739-8526, Japan

## Abstract

Because the functions of polar materials are governed primarily by their polarization response to external stimuli, the majority of studies have focused on controlling polar lattice distortions. In some perovskite oxides, polar distortions coexist with nonpolar tilts and rotations of oxygen octahedra. The interplay between nonpolar and polar instabilities appears to play a crucial role, raising the question of how to design materials by exploiting their coupling. Here, we introduce the concept of ‘polarization twist’, which offers enhanced control over piezoelectric responses in polar materials. Our experimental and theoretical studies provide direct evidence that a ferrielectric perovskite exhibits a large piezoelectric response because of extended polar distortion, accompanied by nonpolar octahedral rotations, as if twisted polarization relaxes under electric fields. The concept underlying the polarization twist opens new possibilities for developing alternative materials in bulk and thin-film forms.

Structural diversity of perovskite oxides offers exciting opportunities for exploring the functions of dielectrics^1^, ferroelectrics^2^ and multiferroics^3^,^4^. Competition between different order parameters leads to a wide variety of phases^5^,^6^,^7^ and evokes enhanced responses to external stimuli, such as stress, magnetic and electric fields^8^,^9^,^10^. Control over the electronic and lattice degrees of freedom can yield the desired properties and provide access to emergent physical phenomena. Therefore, understanding and manipulating the interplay between structural instabilities resulting in distinct types of lattice distortions provide a framework for designing materials with tailored functionalities.

A technologically important distortion has a polar nature that induces ferroelectric polarization. The polar displacement, arising from the off-centring of cations with respect to oxygen octahedra, induces spontaneous polarization (*P*_s_). Because the behaviour of the *P*_s_ vector under electric fields governs ferroelectric-related properties, many studies have investigated the static and dynamic responses of the polar distortion. Recently, detailed structural analyses and theoretical calculations of lead-based ferroelectrics have revealed that the *P*_s_ vector rotates under electric fields, inducing a large piezoelectric response^11^,^12^,^13^,^14^,^15^. The mechanism of the polarization rotation is closely associated with a flattening of the free-energy profile, in which several ferroelectric instabilities compete with each other. The phase diagram includes rhombohedral and tetragonal regions, between which a monoclinic phase exists and acts as a bridging scaffold. The monoclinic phase provides distinct pathways in the free-energy profile, yielding the polarization rotation under weak fields.

Regarding the response of ferroelectrics to external fields, the majority of studies have focused on the polarization and strain originating from the polar distortion. However, some perovskites also exhibit nonpolar instabilities related to tilts and rotations of oxygen octahedra. For hexagonal manganite multiferroics, a nonpolar octahedral rotation with a double-well energy potential that constitutes the primary order parameter causes a ferroelectric polarization with a single-well potential^16^,^17^. This nonpolar rotation strongly affects the electrical properties^18^. Recently, structural asymmetry in layered perovskite heterojunctions was found to induce nonpolar octahedral rotations, resulting in interface ferroelectricity^19^,^20^. The nonpolar octahedral rotations derived from the condensation of zone-boundary phonon modes significantly affect the polarization response. However, studies investigating the interplay between nonpolar and polar instabilities have yet to fully explore novel functions of polar materials.

In this study, we introduce the concept of ‘polarization twist’, which enables enhanced control over piezoelectric responses by exploiting the coupling between nonpolar and polar instabilities. We select a solid solution composed of bismuth sodium titanate (Bi_0.5_Na_0.5_)TiO_3_ (BNT) and barium titanate BaTiO_3_ (BT) as a model polar material because the BNT–BT system contains various structural features^21^,^22^,^23^,^24^. *In situ* X-ray diffraction (XRD) analysis using high-energy synchrotron radiation demonstrates that electric fields induce an extended polar displacement associated with nonpolar octahedral rotations in ferrielectric crystals, as if twisted polarization relaxes and stretches. The twisted polarization creates a large piezoresponse and eventually becomes a ferroelectric polarization. Our simulations based on density functional theory (DFT) and phenomenological theory show that this concept stems from a structural coupling between nonpolar octahedral rotation and polar distortion.

## Results

### Strain and polarization properties of ferrielectric single crystals

**Figure 1:**
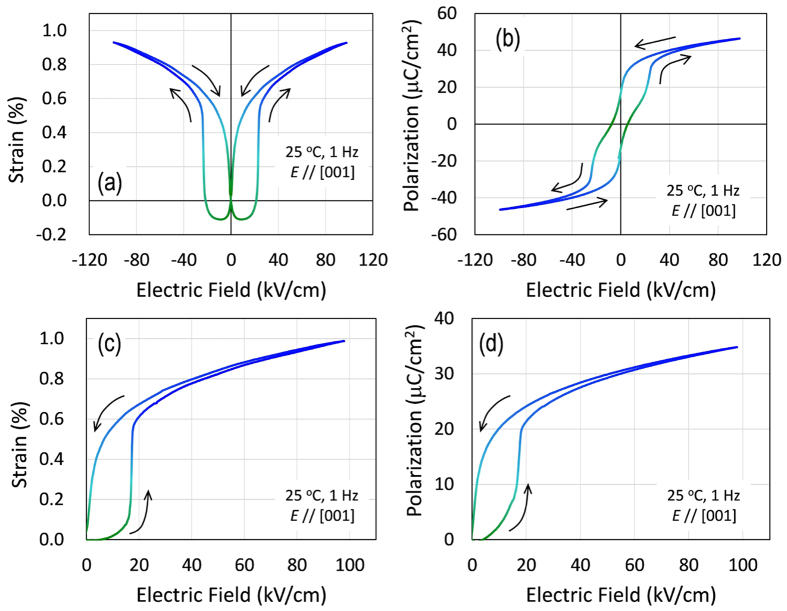
Strain and polarization hysteresis curves with a sharp jump. Under a bipolar (**a**,**b**) and unipolar (**c**,**d**) electric field along the [001] direction, the BNT–7%BT single crystals exhibit peculiar strain (**a**,**c**) and polarization (**b**,**d**) curves with jumps at approximately 20 kV/cm.

Figure 1 shows the strain (*S*) and polarization (*P*) properties under an electric field (*E*) applied along the [001] direction. The crystals present a butterfly-type *S-E* curve (Fig. 1a) with large jumps at *E* = ±20 kV/cm and a double-hysteresis-like *P-E* loop (Fig. 1b). Unlike antiferroelectrics with a remanent polarization (*P*_r_) of zero^25^, the crystals have a certain *P*_r_. This apparent *P*_r_ associated with the pinched hysteresis suggests that the crystals have the ferrielectric phase in space group *P*4*bm* at *E* = 0, as revealed for BNT–7%BT powders by neutron diffraction structural analysis^26^.

In Fig. 1c,d, we display the *S-E* and *P-E* curves measured under unipolar electric fields. Prior to the measurements, an *E* of 100 kV/cm (*E*_poling_) was applied for the poling treatment, and the unipolar *E* was applied in the same direction as *E*_poling_. The *E*-induced *S* with increasing *E* (Fig. 1c) features an extremely large hysteresis with an abrupt jump at *E* = 17 kV/cm, and the *S* value reaches 1.0% at *E* = 100 kV/cm. In the high *E* region of 60–100 kV/cm, the *S-E* curve exhibits a linear response. A decrease in *E* below *E* = 20 kV/cm diminishes *S* substantially, decreasing it to 0% at *E* = 0 kV/cm. We observed a similar hysteresis in the *P-E* curve (Fig. 1d). These *E*-induced properties associated with the abrupt jumps in *S* and *P* are repeatedly identified.

### Structural analyses via *in situ* synchrotron radiation XRD

**Figure 2:**
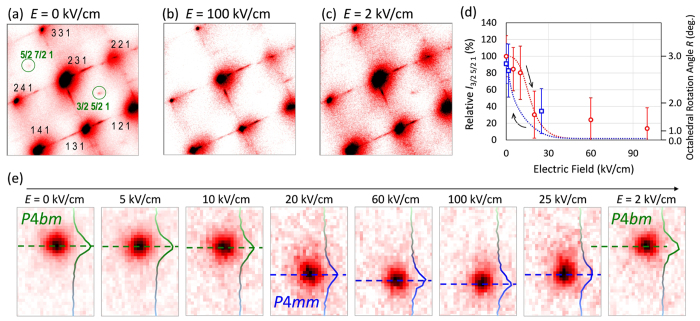
Evolution of the fundamental and superlattice reflections of the BNT–7%BT single crystals under unipolar electric fields observed by the *in situ* SR-XRD measurements. (**a**–**c**) Diffraction patterns observed under an applied electric field (*E*) along the [001] direction: (**a**) *E* = 0 kV/cm (the poled state), (**b**) *E* = 100 kV/cm and (**c**) *E* = 2 kV/cm (with decreasing *E*). (**d**) Variation in the relative intensity of the superlattice 3/2 5/2 1 reflection as a function of *E*. (**e**) Diffraction patterns of the fundamental 1 3 8 reflection recorded at each *E*. Cross-sectional profiles of the 1 3 8 reflection are shown in **e** along the vertical direction in the same pixel.

Figure 2 shows the results of the *in situ* synchrotron radiation XRD (SR-XRD) measurements under electric fields. In the poled state at *E* = 0 kV/cm (Fig. 2a), all diffraction spots arise from a ferrielectric tetragonal structure in space group *P*4*bm*^22^,^26^,^27^. Among the fundamental *hkl* reflections from the pseudocubic cell, we detect 1/2{*o o e*} superlattice reflections, where *o* denotes an odd number and *e* an even number. These superlattice reflections are caused by the tilting of the TiO_6_ octahedra of the *P*4*bm* phase. The crystals in the virgin state present the same diffraction pattern including the superlattice reflections (Supplementary Note 1). We verify that the crystals belong to the tetragonal *P*4*bm* phase in both the virgin and poled states. The *P*4*bm* structure is characterized by a small *P*_s_ along the *c* axis, which is associated with the octahedral tilting around the *c* axis; the details are explained in Supplementary Notes 2 and 3.

The superlattice reflections exhibit a substantial decrease in intensity under electric fields and disappear at *E* = 100 kV/cm (Fig. 2b). The structural analyses of the SR-XRD patterns reveal that the crystals under *E* = 100 kV/cm have a single phase of ferroelectric tetragonal *P*4*mm* without octahedral tilting. We note that the application of *E* induces the phase transition from the tetragonal *P*4*bm* (ferrielectric) to the tetragonal *P*4*mm* (ferroelectric) phase.

When the external field is decreased from 100 kV/cm to 2 kV/cm, the superlattice reflections reappear (Fig. 2c), and then the crystals return to the initial state at *E* = 0 kV/cm. Figure 2d shows the relative intensity of the 3/2 5/2 1 (superlattice) reflection as a function of *E* obtained by the *in situ* measurements. To compensate for the time-dependent change in X-ray strength, the reflection intensity in Fig. 2d is normalized to the background intensity around the 3/2 5/2 1 spot. The right axis indicates the rotation angle of the TiO_6_ octahedra, which is approximately proportional to the square root of the 3/2 5/2 1 reflection intensity. Here, we adopt the rotation angle of 3.0° at *E* = 0 kV/cm determined for BNT–7%BT powders by neutron diffraction structural analysis^26^. With increasing *E*, the superlattice intensity decreases markedly and reaches 14 ± 25% at *E* = 100 kV/cm. With decreasing *E*, a significant intensity cannot be detected down to 30 kV/cm. The superlattice intensity recovers below *E* = 20 kV/cm and returns to its initial value (91 ± 24%) at *E* = 0 kV/cm. We found the following experimentally: the application of an *E* exceeding 20 kV/cm stabilizes the *P*4*mm* phase, and the *E*-induced *P*4*mm* phase returns to the *P*4*bm* phase at *E* = 0 kV/cm. These results clarify that the *E*-induced *P*4*bm*-*P*4*mm* phase transition has a reversible switching path, as discussed in detail below.

We found that both the superlattice reflections and the fundamental *hkl* reflections vary under electric fields. In Fig. 2e, we show the evolution of the 1 3 8 reflection recorded in the same region of the imaging plate as a function of *E*. The application of *E* yields neither a split nor a satellite of the fundamental reflections and only leads to a shift of the peak positions. The line profile beside each spot in Fig. 2e displays the cross-sectional intensity in the vertical direction. The 1 3 8 reflection exhibits a sudden shift between *E* = 10 kV/cm and 20 kV/cm with increasing *E*, which is attributed to the transition from the *P*4*bm* phase to the *P*4*mm* phase. The *P*4*mm* phase persists when *E* > 20 kV/cm with increasing and decreasing electric fields. When *E* is below 25 kV/cm, the spot moves to the line of the *P*4*bm* phase and finally returns to its initial position at *E* = 0 kV/cm. These results show that neither a multidomain state nor a mixed-phase appears in our crystals within the resolution of the SR-XRD measurements.

### Unit cell deformation under electric fields

**Figure 3:**
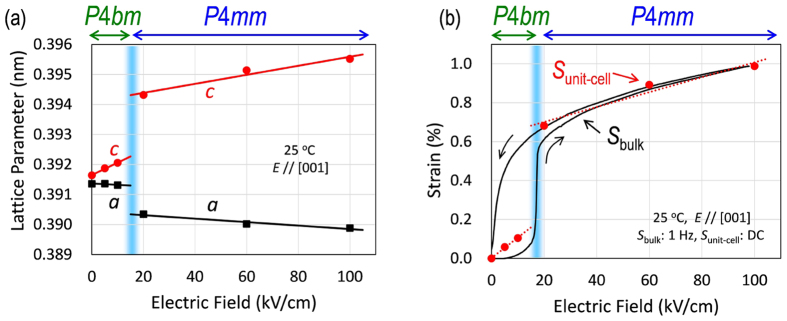
Unit-cell deformation of the BNT–7%BT single crystals under electric fields along the [001] direction (on increasing) evaluated via *in situ* SR-XRD. (**a**) Lattice parameters (*a* and *c*), (**b**) strain of the unit-cell (*S*_unit-cell_) calculated from the parameter *c* in **a** and the strain curve measured for the bulk crystals (*S*_bulk_, see Fig. 1c).

Figure 3a displays the lattice parameters of the pseudocubic unit cell as a function of increasing *E*. The crystal lattice expands along the *c* axis (|| *E*), accompanied by a shrinkage in the *a-a* plane (⊥ *E*) because of the converse piezoelectric effect. The *P*4*bm* phase has a small tetragonal distortion of *c*/*a* = 1.0007 at *E* = 0 kV/cm, which is consistent with the results obtained for BNT–7%BT powders (*c/a* = 1.0003) via neutron diffraction^26^. The crystals feature an abrupt elongation along the *c* axis at *E* = 17 kV/cm. The *P*4*bm*-*P*4*mm* phase transition leads to a discontinuous change not only in the lattice parameters but also in the tetragonal distortion. Our structural analyses demonstrate that the crystals exhibit an *E*-induced reversible phase transition between the *P*4*bm* and *P*4*mm* phases.

Figure 3b shows the strain of the unit cell (*S*_unit-cell_) along the *c* axis (|| *E*) calculated from the lattice parameter *c* (Fig. 3a). The *S*_unit-cell_ values coincide well with the strain of the bulk crystals (*S*_bulk_, Fig. 1c) measured with increasing *E*. It is clear that the macroscopic response of *S*_bulk_ originates from the microscopic deformation of the unit cell, i.e., the change in *S*_unit-cell_ caused by the application of *E*. The quantitative agreement between *S*_bulk_ and *S*_unit-cell_ provides direct evidence that the large jump in *S*_bulk_ at *E* = 17 kV/cm stems from the *E*-induced phase transition from the *P*4*bm* phase (*c/a* ≤ 1.002) to the *P*4*mm* phase (*c/a* ≥  1.010).

## Discussion

**Figure 4:**
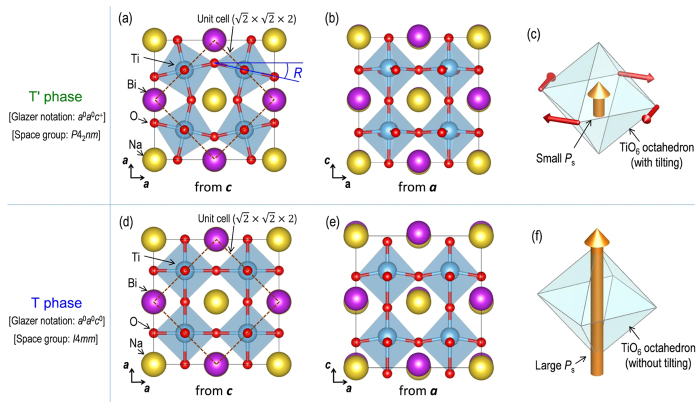
Two tetragonal phases obtained by the structural optimizations via DFT calculations. Crystal structures of the T’ phase (**a**–**c**) and the T phase (**d**–**f**): panels **a** and **d** are projected from the *c* axis, panels **b** and **e** from the *a* axis. Panels **c** and **f** schematise the structural features regarding polarization and octahedral tilting.

We investigated the *P*4*bm* (ferrielectric)-*P*4*mm* (ferroelectric) phase transition induced by electric fields using density functional theory (DFT) calculations. The structural optimizations revealed that BNT with a rock-salt-like A-site ordering forms two types of tetragonal structures with comparable energies. The detailed methodology is described in the Calculation Methods section. Figure 4 depicts the crystal structures of the two tetragonal phases. One exhibits a weak-polar structure associated with octahedral tilting around the *c* axis (Fig. 4a,b), which is denoted by the ‘T’ phase’. Because the T’ phase has an octahedral tilt involving a small *P*_s_ along the *c* axis as schematised in Fig. 4c (that is, it features the *P*4*bm* structure), we can regard the T’ phase as the *P*4*bm* phase. The other has a strong-polar structure exhibiting a large *P*_s_ along the *c* axis without octahedral tilting (Fig. 4d–f), which is called the ‘T phase’. Because the T phase has the essential structural elements of the *P*4*mm* phase and the symmetry of its TiO_6_ octahedra can be approximated in space group *P*4*mm*, the T phase corresponds to the *P*4*mm* phase.

**Figure 5:**
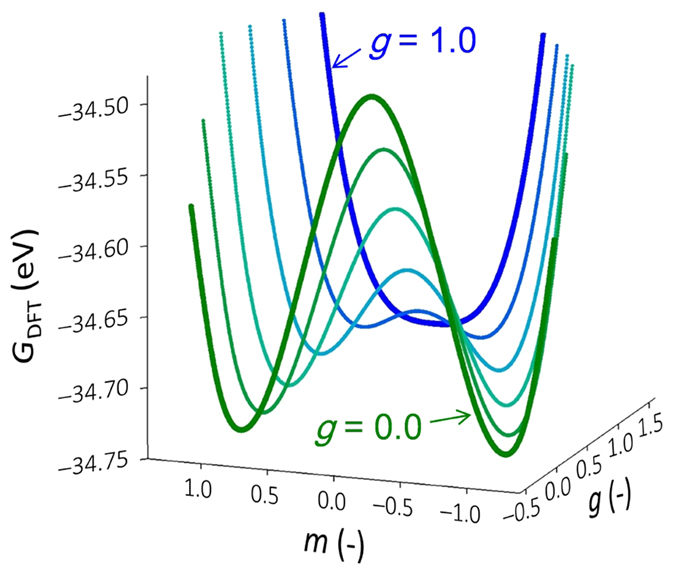
Free-energy profiles obtained by the DFT calculations. *G*_DFT_ profiles (zero electric field) in the two-dimensional (*g*, *m*) subspace, where g and *m* represent the polar displacement and the rotational degree of the oxygen octahedra. The curves are traced with respect to *m* from *g* = 0 (green) to *g* = 1 (blue). The double minima at *g* < 1 correspond to the T’ phase, whereas the single minimum at *g* = 1 shows the T phase.

Here, we discuss the total free energy (*G*_DFT_) obtained by the DFT calculations. The formula for *G*_DFT_ is expressed as


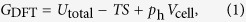


where *U*_total_ denotes the total energy of the perovskite unit cell, *TS* is the entropy term, and *V*_cell_ is the cell volume. We employed zero-temperature DFT calculations; thus, *TS* = 0. We define two structural parameters, *g* and *m*, representing the crystallographic features of the T’ and T phases: *g* is the degree of the polar displacement leading to *P*_s_ along the *c* axis, and *m* is the degree of the octahedral rotation around the *c* axis. The fractional coordinates of the atoms in the unit cell can be described by the parameters (*g*, *m*) and are defined such that the T’ and T phases (Fig. 4) are located at (*g*, *m*) = (0, 1) and (1, 0), respectively. The detailed definitions of these parameters are provided in the Calculation Methods section. Figure 5 shows the *G*_DFT_ profiles as functions of *g* and *m*. For each profile, the parameter *g* (the polar displacement) is fixed, and the parameter *m* (the octahedral rotation) is varied. At *g* = 0, the *G*_DFT_ profile shows a double-minimum potential with respect to *m*. The potential minima correspond to the T’ phase exhibiting the octahedral rotation, the degree of which is defined as *m* = 1. Increasing *g* from 0 to 1 lowers the potential height between the minima, and the *m* values at the minima approach zero. At *g* = 1.0 (that is, for the T phase with the large polar displacement), the *G*_DFT_ profile presents a single-minimum potential. We note that the *g* and *m* parameters exhibit a trade-off relationship. Large values of both *g* and *m* cannot coexist; thus, a large *g* is achieved by sacrificing *m* and vice versa.

To gain further insight into the relation between the T’ and T phases, we examine the free-energy potential based on the phenomenological Landau-Ginzburg-Devonshire (LGD) theory^28^. We start from a free-energy function of *G*_LGD_ to express a ferrielectric-ferroelectric phase transition by


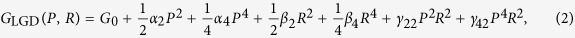


where two order parameters, *P* and *R*, are included: *P* is the net polarization along the polar axis, *R* denotes the degree of the octahedral rotation around the polar axis, and the rests are the independent parameters. By choosing appropriate parameters (see Supplementary Note 4), we can obtain the *G*_LGD_ potential involving two local minima at (*P*, *R*) = (*P*_a_, *R*_a_) and (*P*_b_, 0) in the *P* ≥ 0 and *R* ≥ 0 region, where *P*_a_ and *P*_b_ indicate small and large polarizations, respectively, and *R*_a_ represents the degree of the octahedral rotation. Therefore, we can consider the former minimum as the T’ phase (a small *P*_s_ with octahedral tilting) and the latter as the T phase (a large *P*_s_ without octahedral tilting).

Next, we introduce the following relations to correlate the order parameters (*P* and *R*) of the *G*_LGD_ function with the structural parameters (*g* and *m*):


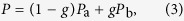



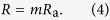


**Figure 6:**
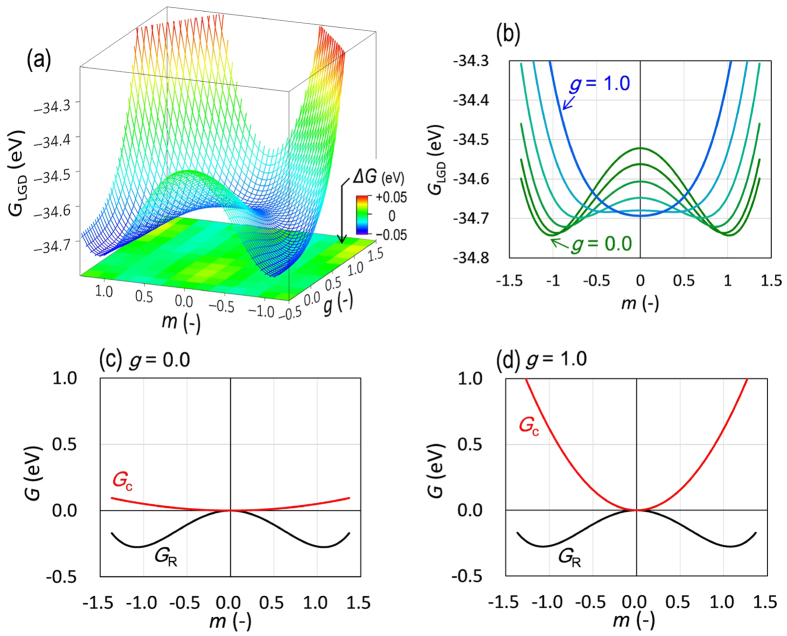
Free-energy features obtained by the LGD theory. (**a**) Free-energy (*G*_LGD_) surface in the two-dimensional (*g*, *m*) subspace and (**b**) cross-sectional profiles as a function of *m* correlated with the order parameter *R*. (**c**,**d**) Contributions of the octahedral rotation term (*G*_*R*_) and the coupling term (*G*_c_) as a function of *m* at a fixed *g*: (**c**) *g* = 0.0 and (**d**) *g* = 1.0.

These definitions result in the *G*_LGD_ potential exhibiting two potential minima at (*g*, *m*) = (0, 1) and (1, 0). Figure 6a shows the *G*_LGD_ landscape obtained by fitting the *G*_LGD_ function to the *G*_DFT_ potential. The difference between these values can be smaller than 0.05 eV, especially by virtue of the coupling terms (*γ*_22_*P*^2^*R*^2^ + γ_42_*P*^4^*R*^2^) in Eq. 2 (see Supplementary Note 4). In addition, a reasonable relation of the polarizations, 0 < *P*_a_ < *P*_b_, is realized for the T’ and T phases. Figure 6b represents the cross-sectional profiles of the *G*_LGD_ surface at various *g* values. The overall features of the *G*_DFT_ profile (Fig. 5) are well reproduced by the *G*_LGD_ theory. The agreement between the two calculations (Figs 5 and 6a) confirms that the LGD theory with the two order parameters (*P* and *R*) can describe the T’-T phase transition in a simple analytical manner.

The calculations based on the LGD theory enable us to identify the structural factor dominating the T’-T transition. Figure 6c,d displays the energy curves with respect to *m*, corresponding to the octahedral rotation (*G*_*R*_) and the polarization-rotation coupling (*G*_c_) in the *G*_LGD_ function: *G*_*R*_ = *α*_2_*R*^2^/2 + *α*_4_*R*^4^/4 and *G*_c_ = *γ*_22_*P*^2^*R*^2^ + *γ*_42_*P*^4^*R*^2^. The sum of the two terms, *G*_*R*_ + *G*_c_, determines the overall shape of the *G*_LGD_ landscape. Because *G*_*R*_ is independent of *g*, *G*_c_ is decisive in the *G*_LGD_ function. The *γ*_22_ and *γ*_42_ coefficients are positive; therefore, the *G*_c_ term yields a positive quadratic variation with *m*. At *g* = 0 (Fig. 6c), *G*_c_ plays no role in *G*_LGD_ because of the small *P* (=*P*_a_), and hence, the *G*_LGD_ function at *g* = 0 directly reflects the double-minimum *G*_*R*_ character. At *g* = 1.0 (Fig. 6d), the large *G*_c_ quadratic negates the double minimum in *G*_*R*_ and changes the *G*_LGD_ function to the single-minimum potential. The LGD theory demonstrates that the *g*-dependent *G*_c_ character governs the free-energy feature in the T’-T phase transition. The *G*_c_ term (namely, the polarization-rotation coupling), dominates the competing energy relation between the T’ and T phases.

**Figure 7:**
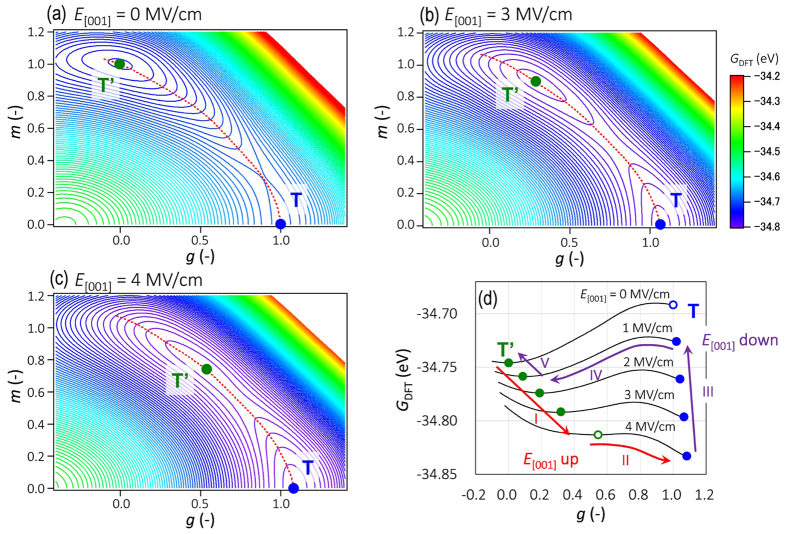
Free-energy maps obtained by DFT calculations under electric fields. The *G*_DFT_ maps in the (*g*, *m*) subspace under electric fields (*E*_[001]_) of (**a**) 0 MV/cm, (**b**) 3 MV/cm and (**c**) 4 MV/cm; *g* denotes the polar displacement, and *m* denotes the rotational degree of the oxygen octahedra. Red dashed lines in the maps indicate the energy valley passing through the two local minima corresponding to the T’ and T phases. (**d**) *G*_DFT_ profiles along the energy valley at each *E*_[001]_.

We then analyse the detailed structural variation under electric fields using DFT calculations. The influence of *E* on the electronic energy *U*_total_ in Eq. 1 can be included by the perturbation expansion after the discretization (PEAD) approach^29^ using the macroscopic polarization, as defined in the ‘modern theory of polarization’^30^,^31^. Figure 7 shows the *G*_DFT_ maps in the (*g, m*) subspace under an electric field (*E*_[001]_) applied along the [001] direction (the *c* axis). The red dashed line depicts the energy valley exhibiting the switching path between the T’ and T phases. At *E* = 0 MV/cm (Fig. 7a), the *G*_DFT_ map has a deep minimum at (*g, m*) = (0, 1) and a shallow minimum at (*g, m*) = (1, 0). The former corresponds to the T’ phase, which is characterized by a small polarization with apparent octahedral tilting. The latter is equivalent to the T phase, exhibiting a large polarization without octahedral tilting.

Figure 7d presents the *G*_DFT_ variations along the energy valley as a function of *g*. When an *E*_[001]_ is applied, the T’ phase moves towards the T phase (denoted by I). Moreover, *E*_[001]_ lowers the energy barrier from the T’ to T phase (Δ*G*_T’→T_). The Δ*G*_T’→T_ value at *E*_[001]_ = 3 MV/cm (Fig. 7b) is 9.0 meV, which is considerably less than 56 meV at *E* = 0 MV/cm (Fig. 7a). Given that *E*_[001]_ reaches 4 MV/cm (Fig. 7c), Δ*G*_T’→T_ is as small as 1.3 meV, which is one order of magnitude smaller than the thermal energy at room temperature (*k*_B_*T*_RT_): 26 meV. The T’ phase exceeds this small Δ*G*_T’→T_ by the thermal energy and eventually transits to the T phase under such high fields (II in Fig. 7d).

With decreasing *E*_[001]_, the T phase remains stabilized in the high-*E*_[001]_ region with a slight decrease in its polar displacement (III in Fig. 7d), and the energy barrier from the T to T’ phase (Δ*G*_T→T’_) is lowered. The Δ*G*_T→T’_ value decreases to 4.5 meV at *E* = 1 MV/cm, which is much smaller than *k*_B_*T*_RT_. This change results in a reverse transition from the T to T’ phase (IV in Fig. 7d). As *E*_[001]_ decreases further, the T’ phase returns to its initial state (*g*, *m*) = (0, 1) at *E* = 0 (V in Fig. 7d). Our DFT calculations taking electric fields into account show that the switching path for the reversible phase transition lies between the T’ and T phases, as identified by the experimental results (Fig. 2).

**Figure 8:**
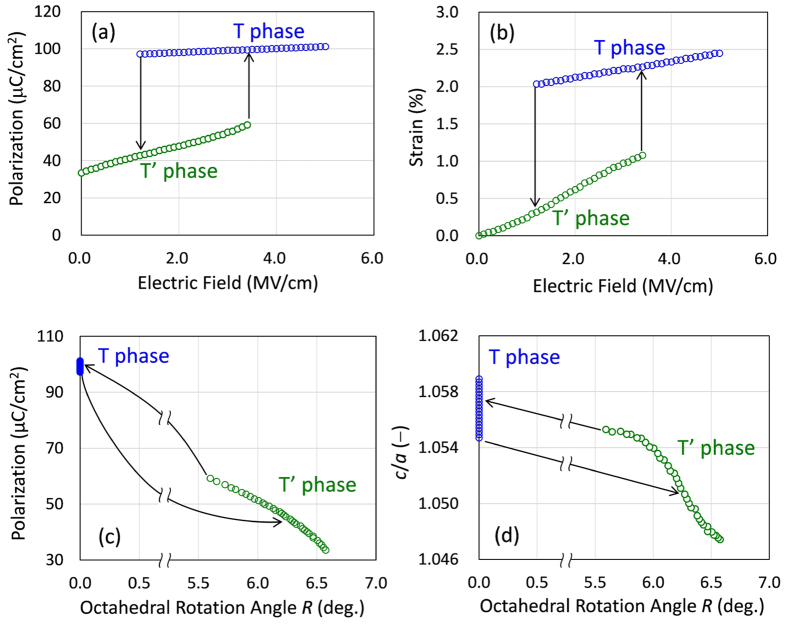
Polarization and strain hysteresis loops accompanied by octahedral rotations obtained by DFT calculations under a unipolar *E*_[001]_. (**a**) Polarization-electric field (*P*-*E*) and (**b**) strain-electric field (*S*-*E*) curves of the T and T’ phases calculated from the unit-cell structures at the local minima in the *G*_DFT_ maps. Evolutions of (**c**) polarization and (**d**) tetragonal distortion (*c*/*a*) with respect to the octahedral rotation angle (*R*) under unipolar *E*_[001]_.

Figure 8a,b displays the polarization (*P*_DFT_) and strain of the unit cell (*S*_DFT_) as a function of *E*_[001]_ obtained by our *G*_DFT_ calculations. The *S*_DFT_ values are estimated from the unit-cell structures at the local minima moving along the energy valley in the (*g*, *m*) space. We assume here that the transition between the T’ and T phases occurs once the barrier of Δ*G*_T’→T_ or Δ*G*_T→T’_ is below 5 meV. The *P*_DFT_ variation (Fig. 8a) shows that the crystal lattice undergoes a reversible phase transition with an apparent hysteresis. As *E*_[001]_ increases, the T’ phase with a small *P*_DFT_ changes to the T phase with a large *P*_DFT_ at a threshold *E*_[001]_. As *E*_[001]_ decreases, the *P*_DFT_ value remains large and then drops sharply because of the reverse transition from the T to T’ phase. The *S*_DFT_ curve (Fig. 8b) also exhibits hysteresis with abrupt jumps with both increasing and decreasing *E*_[001]_. The jumps in *S*_DFT_ are attributed to the phase transitions between the T’ and T phases, as can be also seen in the *P*_DFT_ variation. The polarization and strain curves simulated by the DFT calculations are in qualitative agreement with the experimental results (Fig. 1).

Here, we discuss the ferrielectric features under unipolar electric fields and the associated piezoelectric response. Our experiments demonstrate that an application of *E* enhances *P* (Fig. 1d) and reduces the rotation angle (*R*) of the TiO_6_ octahedra (Fig. 2d). Our DFT calculations also show that an *E*_[001]_ application increases the polarization (Fig. 8c) and the tetragonality (*c*/*a*, Fig. 8d) accompanied by a decrease in *R*. It is worth noting that the free-energy surface of the T’ phase is incredibly flat compared with that of the T phase (see Fig. 7 and Supplementary Note 5). With increasing *E*, the T’ phase moves substantially in the (*g*, *m*) subspace along the energy valley with a gentle slope. An external field induces an extended polar displacement with a suppressed rotational distortion of the oxygen octahedra, where the *P*_s_ vector behaves as if the twisted polarization relaxes and stretches. The polarization twist yields a linear increase in *S*_DFT_ with *E*_[001]_ (Fig. 8b) because of the converse piezoelectric effect in the low-field region. We observed this behaviour also in the *S*_unit-cell_ data from the SR-XRD analyses (Fig. 3b). The piezoelectric strain constant of the ferrielectric *P*4*bm* phase estimated from the slope of *S*_unit-cell_ is as high as 1,000 pm/V, which is, to our knowledge, the highest for lead-free ferroelectrics. The higher strain constant of the T’ phase is also validated by the DFT calculations (Fig. 8b). The superior piezoelectric character in the ferrielectric phase stems from the flat free-energy profile unique to the polarization twist compared with the polarization extension in the ferroelectric phase. We expect that ferrielectrics involving octahedral rotation potentially possesses enhanced piezoelectric properties compared with ferroelectrics. Thus, we identify the polarization twist as a new type of piezoresponse, in addition to polarization rotation^11^,^12^ and polarization extension^32^. Exploiting the interplay between nonpolar and polar instabilities in ferrielectrics is expected to provide a new degree of freedom for developing high-performance perovskite materials for use in bulk and thin-film forms.

## Methods

### Crystal growth and electric measurements

Single BNT–7%BT crystals were grown by a top-seeded solution growth method at a high oxygen pressure (*P*o_2_ = 0.9 MPa)^33^,^34^. First, BNT–BT powders prepared via a solid-state reaction were mixed with a flux composed of Bi_2_O_3_, NaF and BaCO_3_ and placed in a platinum crucible. The mixture was soaked at 1,100 °C for 4 h, slowly cooled to 1,070 °C at a rate of −2 °C/h, and then cooled to room temperature. The details of the crystal growth are described in refs 34, 35, 36. We confirmed the chemical composition of BNT–7%BT via X-ray fluorescence and inductively coupled plasma-atomic emission spectrometry.

The crystals were cut into plates with thicknesses of 0.2 mm. The axis normal to the crystal plates was along the <100> direction. Gold electrodes were sputtered on both cut surfaces of the platelet crystals. We investigated the electrical properties along the [001] direction. Strain properties were measured using a laser Doppler displacement meter.

### SR-XRD measurements

We performed the SR-XRD analyses with a transmission geometry using a large cylindrical two-dimensional imaging plate camera at BL02B1 in the SPring-8 synchrotron radiation facility^37^,^38^. We adopted a high SR energy of 35 keV [wavelength: 0.035313(15) nm] such that the X-ray could penetrate through the crystals and a high-angle diffraction pattern could be observed. The X-ray beam incident on the crystals was 150 μm in diameter, and the measurement temperature was 25 °C. To investigate the intrinsic response of the unit cell with respect to *E*, we conducted *in situ* measurements of the diffraction patterns under the application of a static *E* parallel to the [001] direction. We obtained four X-ray oscillation photographs with an oscillation angle of 10° at different angles between the (100) face and the incident X-ray beam at each electric field. We refined the lattice parameters via the least-squares method using approximately 150 peaks of the *hkl* reflections (*d* ≥ 0.04 nm). We analysed the diffraction data by imposing the following structural constraints on the lattice parameters: *a* = *b* and *α* = *β* = *γ* = 90°.

### Calculation methods

First-principles calculations based on density functional theory (DFT) were performed to investigate the *E*-induced *P*4*bm* (T’)-*P*4*mm* (T) phase transition. While BNT and its related materials do not exhibit a long-range ordering of the A-site cations^39^,^40^, we need to apply a periodic arrangement of Bi and Na to the BNT model structure for the DFT calculations. We chose pure BNT with a rock-salt-like A-site ordering^40^,^41^ as a model substance. First, we constructed the supercell consisting of 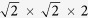 perovskite, which is defined as the T_super_ cell. The fractional coordinates of the constituent atoms are listed in Table 1. The T_super_ cell contains two types of atomic displacements: polar displacement along the *c* axis (*z*_*i*_) yielding *P*_s_ and nonpolar displacement (*x*_*i*_) leading to a tilting of the TiO_6_ octahedra around the *c* axis (*i* is the index of atoms). The T and T’ structures can be described by the T_super_ cell as follows: the T phase has a relatively large *z*_*i*_ with *x*_*i*_ = 0, whereas the T’ phase features a small *z*_*i*_ with a certain degree of *x*_*i*_. The T phase constructed in the T_super_ cell has *I*4*mm* symmetry, and the T’ phase in the T_super_ cell has *P*4_2_*nm* symmetry. These symmetries are lower than those of the actual *P*4*mm* and *P*4*bm* phases for the T phase and T’ phase, respectively, due to the A-site ordering adopted in the T_super_ cell: i.e., *I*4*mm* is a subgroup of *P*4*mm* while *P*4_2_*nm* is a subgroup of *P*4*bm*.

The experimental results presented in Fig. 3 show that the free energy (*G*) of the T’ phase is small but comparable to that of the T phase at *E* = 0. We adopted a hydrostatic pressure (*p*_h_) of 6.0 GPa in our DFT calculations, at which the *G* feature that the T’ phase is stabilized compared to the T phase (metastable) is satisfied. The methodology is summarized in Supplementary Note 6. The structural optimization of the T_super_ cell at *p*_h_ = 6.0 GPa leads to the T’ phase, in which the fractional coordinates are denoted by *z*_*i*_(T′_*E*=0_) and *x*_*i*_(T′_*E*=0_). The optimization of the T_super_ cell under the constraint of *x*_*i*_ = 0 results in the T phase, and its coordinate is defined as *z*_*i*_(T_*E* = 0_). We confirmed that the optimized structure of the T phase possesses a larger tetragonal distortion (*c*/*a*) than the T’ phase (*c*/*a* = 1.047 for the T’ phase and 1.053 for the T phase).

Here, we introduce the two-dimensional subspace (*g*, *m*) representing the crystal structures with various degrees of *P*_s_ along the *c* axis and of the TiO_6_ tilting around the *c* axis. The parameter *g* indicates the degree of the polar displacement, which is expressed as


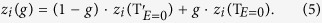


The parameter *m* expresses the degree of the TiO_6_ tilting as


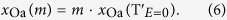


In the (*g*, *m*) subspace at *E* = 0, the T phase is present at (*g*, *m*) = (1, 0), and the T’ phase is located at (*g*, *m*) = (0, 1). If the parameters *g* and *m* are given, the fractional coordinates of the T_super_ cell can be specified according to Eqs 5 and 6. The structure optimizations of the lattice parameters (*a* and *c*) were performed at *p*_*h*_ = 6.0 GPa with the fixed fractional coordinates at each of the mesh points in the (*g*, *m*) subspace. After the optimizations, these T_super_ cells are used to calculate the free energy *G*_DFT_ in the (*g*, *m*) space according to Eq. 1.

The DFT calculations were performed according to the generalized gradient approximation^42^ using a plane wave basis set, as implemented in the Vienna *ab initio* simulation package (VASP)^43^. We used the projector-augmented wave potentials^44^ with the valence-electron configurations of 5*d*^10^6*s*^2^6*p*^3^ for Bi, 2*p*^6^3*s*^1^ for Na, 3*p*^6^3*d*^2^4*s*^2^ for Ti, and 2*s*^2^2*p*^4^ for O. The Perdew-Burke-Ernzerhof functional modified for solids (PBEsol)^45^ was employed for the exchange-correlation potential. A plane-wave cut-off energy of 520 eV was adopted, and the total energy was converged to less than 10^−5^ eV in all calculations. The Monkhorst-Pack *k*-mesh of 3 × 3 × 2 was adopted for geometrical optimization calculations of the T_super_ cell. The crystal structures obtained by the DFT calculations (Fig. 4) were drawn using the three-dimensional visualization software VESTA^46^.

## Additional Information

**How to cite this article**: Kitanaka, Y. *et al.* Polarization twist in perovskite ferrielectrics. *Sci. Rep.* **6**, 32216; doi: 10.1038/srep32216 (2016).

## Supplementary Material

Supplementary Information

## Figures and Tables

**Table 1:** Site and coordinates of the constituent atoms in the T_super_ cell (space group: *P*4_2_ *nm*) adopted to investigate the T’-T phase transition.

	site	*x*	*y*	*z*
Na	2a	0	0	1/4 + *z*_Na_
Bi	2a	0	0	3/4 + *z*_Bi_
Ti	4b	0	1/2	*z*_Ti_
O_c_	4b	0	1/2	1/4 + *z*_Oc_
O_a1_	4c	1/4 + *x*_Oa_	1/4 + *x*_Oa_	0
O_a2_	4c	3/4 + *x*_Oa_	1/4−*x*_Oa_	0
