# Supplementary material for: Polarization twist in perovskite ferrielectrics
Source: Sci Rep. 2016 Sep 2;6:32216. doi: 10.1038/srep32216 (PMC5009376; doi:10.1038/srep32216)
Supplement: Supplementary Information [file srep32216-s1.pdf]

Supplementary Information

Title: Polarization twist in perovskite ferrielectrics

Authors: Yuuki Kitanaka, Kiyotaka Hirano, Motohiro Ogino, Yuji Noguchi, Masaru Miyayama, Chikako Moriyoshi, and Yoshihiro Kuroiwa

TABLE OF CONTENTS

1. Crystal structural analysis of the BNT–BT crystals in the virgin state
2. Composition phase diagram of the BNT-BT system
3. Polarization configuration in the ferrielectric *P*4*bm* phase and analogy with antiferroelectric perovskites
4. Formalism of the LGD function for the T’-T phase transition
5. LGD calculations for the switching path of the T’-T phase transition
6. Construction of the model structures for the DFT calculations

1. Crystal structural analysis of the BNT–BT crystals in the virgin state

Supplementary Figure S1a shows an *in situ* synchrotron radiation XRD (SR-XRD) pattern in the virgin state (without any electrical treatment). We clearly identify the 1/2{*o o e*} superlattice reflections arising from the *P*4*bm* phase. We note that the application of E = 100 kV/cm to the virgin crystals obliterates the superlattice reflections (Supplementary Fig. S1b). The spots observed at E = 100 kV/cm can be assigned to the *P*4*mm* phase with a tetragonal distortion (c/a) of 1.013, where a and c denote the lattice parameters. Provided that the electric field is turned off, this *P*4*mm* phase goes back to the *P*4*bm* phase [Fig. 2(a)]. We found that the *P*4*bm*-*P*4*mm* phase transition is reversibly induced by electric fields not only for the virgin crystals but also for the poled ones. In contrast, Ma *et al.*[1,2] reported that the *P*4*bm* phase observed for BNT–xBT ceramics (x = 5–6%) in the virgin state disappears and turns into a mixed-phase state composed of rhombohedral *R*3*c* and tetragonal *P*4*mm* due to poling treatments. Their structural analyses indicate that the virgin ceramics have the *P*4*bm* structure and that the structural energies of the *R*3*c*, *P*4*mm* and *P*4*bm* phases are comparable. Therefore, the metastable mixed-phase state is established in the form of ceramics, likely due to an inhomogeneous distribution of residual stress evoked by the poling treatments. Our analyses of the *in situ* SR-XRD data provide direct evidence of the reversible transition between the *P*4*bm* and *P*4*mm* phases in our crystals.

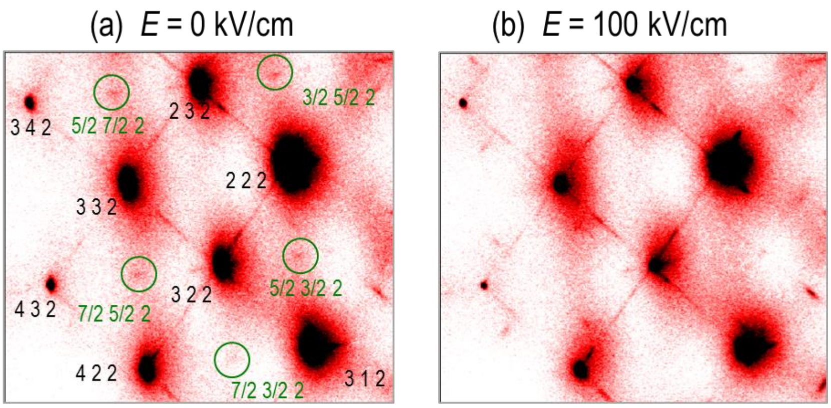

Supplementary Figure S1. SR-XRD patterns of the BNT–7 %BT single crystals (**a**) in the virgin state and (**b**) at an electric field (E) of 100 kV/cm.

2. Composition phase diagram of the BNT-BT system

At room temperature, the parent BNT has a ferroelectric structure in monoclinic *Cc* space group[3,4] (approximated as rhombohedral *R*3*c*)[5], which is consistent with our DFT calculations (the ground state of BNT is the *R*3*c* phase; see Supplementary Fig. S6). With increasing temperature, BNT exhibits a phase transition from the low-temperature ferroelectric *Cc* to the intermediate-temperature ferrielectric *P*4*bm* and then to the high-temperature paraelectric $Pm\bar{3}m$ phase. The details of the phase transition of BNT are described in Ref. 5.

The ferrielectric *P*4*bm* phase can be driven not only by temperature but also by solid composition. Supplementary Figure S2 represents the SR-XRD patterns measured at 25 °C for BNT–*x* BT single crystals with different BT compositions (*x*). For x = 4 % (Supplementary Fig. S2a), the superlattice reflections of 1/2{*o o o*} are evident and can be assigned to the monoclinic *Cc* structure, as reported in Refs. 3,4. Because the structural deviation between the *Cc* and *R*3*c* phases is very small, we can consider that the *Cc* phase has a ferroelectric polarization along the pseudocubic [111] direction, with an antiphase octahedral tilt denoted by $a^-a^-a^-$ in the Glazer notation[6]. The P_s value has been reported as 55 μC/cm^2 for x = 4 %[7]. As shown in Fig. 2 and Supplementary Fig. S1, the x = 7 % crystals display the 1/2{*o o e*} superlattice reflections, which are unique to the *P*4*bm* phase[5,8] with a tilt system of $a^0a^0c^+$. In contrast to x = 4 % (*Cc*) and 7 % (*P*4*bm*), the x = 12 % crystals exhibit no superlattice reflections, as shown in Supplementary Fig. S2b, and their spots are assigned to the tetragonal *P*4*mm* structure. The *P*4*mm* phase has a large P_s (60 μC/cm^2 for x = 12 %)[9] along the [001] direction without octahedral tilting. We also confirm the following composition-structure relations: x = 6 % (*P*4*bm*) and x = 8 % (*P*4*mm*).

Supplementary Figure S2c shows the composition phase diagram of the BNT–*x* BT system at 25 °C based on our single-crystal SR-XRD measurements. The monoclinic *Cc* phase is present on the BNT-rich side ($x < 5$ %), whereas the tetragonal *P*4*mm* phase exists on the BT-rich side ($x > 9$ %). We note that the *P*4*bm* phase is stabilized in the limited x range at approximately x = 6–7 %. This phase diagram demonstrates that the x = 7 % crystals (*P*4*bm*) are in the vicinity of the *P*4*mm* phase.

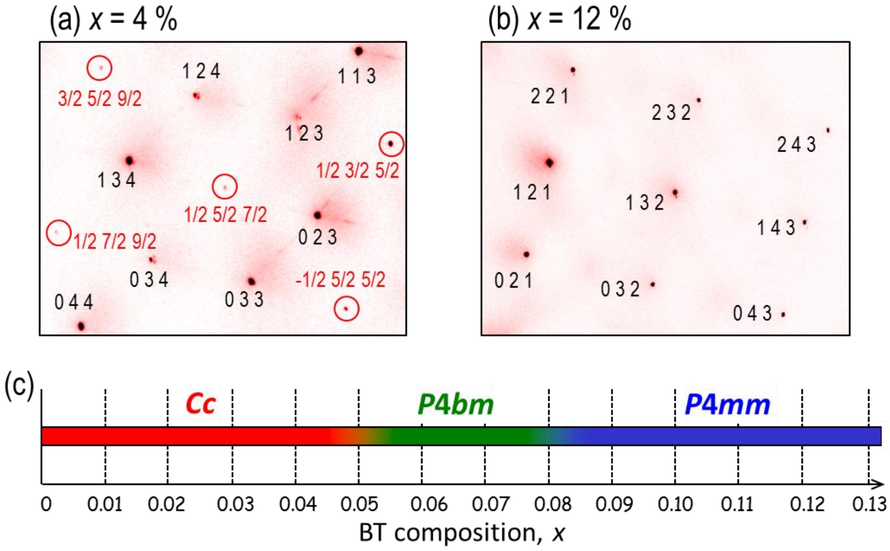

Supplementary Figure S2. (**a, b**) SR-XRD patterns of the BNT–*x* BT single crystals measured at 25 °C: (**a**) $x = 4$ % and (**b**) $x = 12$ %. Circles in **a** indicate the 1/2{*o o o*} superlattice reflections. (**c**) Composition phase diagram of the BNT–BT system at 25 °C.

3. Polarization configuration in the ferrielectric *P*4*bm* phase and analogy with antiferroelectric perovskites

Supplementary Figure S3 depicts the structural and polarization features of the *P*4*bm* phase[8]. Provided that the origin of the polar displacements along the *c* axis is the O_a site, the B-site atoms are displaced along the *c* axis, whereas the A-site and O_c-site atoms show an opposite displacement. The B-site atoms generate an upward polarization (p_1) of 22.4 μC/cm^2 along the *c* axis, whereas the A-site and O_c atoms exhibit a downward polarization (p_2) of −18.5 μC/cm^2. These atomic displacements and the resultant antiparallel polarization configuration lead to the ferrielectric nature with a small spontaneous polarization (P_s) of 4 μC/cm^2 along the *c* axis. The oxygen octahedra in the *P*4*bm* phase exhibit in-phase tilting with an octahedral rotation angle of 3.0 ° along the *c* axis, and the tilt system is denoted by $a^0a^0c^+$.

Here, we consider the analogy of the ferrielectric *P*4*bm* phase with antiferroelectric perovskites represented by $PbZrO_3$[10]. The antiferroelectrics are characterized by a group of coupled phonon modes containing a primary unstable antipolar displacement accompanied by nonpolar distortions, such as oxygen octahedral rotations. The coupling of these unstable modes is symmetry-allowed, and its condensation lowers the energy of the antiferrodistortive structure. In addition, the antiferroelectrics feature the presence of a competing ferroelectric phase, which can be driven by applying electric fields through a first-order phase transition. The distortions found in $PbZrO_3$ can be described by the condensation of a set of coupled modes, most importantly at R and Σ in the Brillouin zone, resulting in zero P_s.

The structural features of the ferrielectric *P*4*bm* phase are explained by a coupling of two phonon modes: one corresponds to the nonpolar M_2^+ irreducible representation at the Brillouin-zone-boundary M point, and the other corresponds to the polar Γ_4^- irreducible representation at the zone-centre Γ point, according to the group theoretical analysis[11,12]. The condensation of these coupled modes yields the ferrielectric structure; the former produces the $a^0a^0c^+$ octahedral tilt, and the latter yields a small P_s arising from the difference of the upward and downward polarizations. In the composition phase diagram (Supplementary Fig. 2c), the *P*4*bm* phase is adjacent to the *P*4*mm* phase, indicating that the *P*4*bm* ferrielectric competes with the *P*4*mm* ferroelectric. The *P*4*bm* phase is very close in energy to the *P*4*mm* phase, which

permits the *P*4*bm*-*P*4*mm* phase transition induced by applying an electric field, as demonstrated by our SR-XRD measurements (see Figs. 2 and 3)

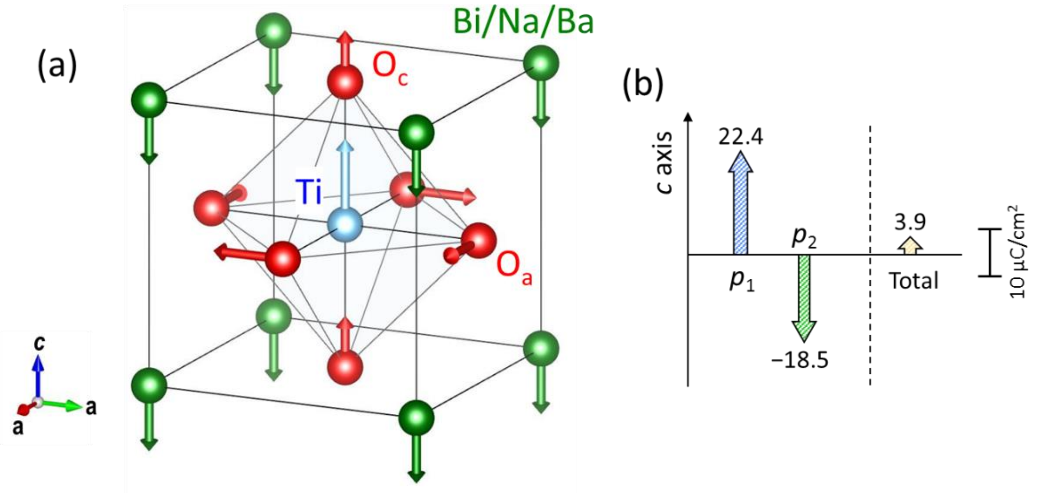

Supplementary Figure S3. Atomic displacements and polarization configuration of the *P*4*bm* phase in BNT–7 %BT[8]. (**a**) Antiparallel displacements of the A- and B-site atoms and the rotation of the oxygen octahedra. Spheres show the atomic sites in the cubic phase and vectors their displacements in the *P*4*bm* phase from the cubic structure. The magnitudes of the vectors are multiplied by 10 of the atomic displacements. The tetragonal distortion (c/a) is as small as 1.0003, which can be approximated to be one. (**b**) Upward and downward polar components leading to the ferrielectric nature of the *P*4*bm* phase with a net polarization of 4 μC/cm^2 along the *c* axis.

4. Formalism of the LGD function for the T'-T phase transition

We investigate the free energies of the T' and T phases based on the phenomenological theory using the two order parameters of polarization P and octahedral rotation R. Here, we formulate the G_{LGD} function as

$$G_{\mathrm{LGD}} = G_0 + G_P + G_R + G_{\mathrm{c}},$$

where G_0 denotes the constant energy, G_P is the polar term, G_R is the octahedral rotation term, and G_{c} is the coupling term between P and R. We write the G_P and G_R functions as

$$G_P = \frac{1}{2}\alpha_2 P^2 + \frac{1}{4}\alpha_4 P^4,$$

$$G_R = \frac{1}{2}\beta_2 R^2 + \frac{1}{4}\beta_4 R^4,$$

where α_2 and β_2 are negative and α_4 and β_4 are positive coefficients. Considering the symmetry constraint of the parent cubic phase, we can describe the coupling term between P and R by

$$G_{\mathrm{c}} = \gamma_{22}P^2R^2 + \gamma_{24}P^2R^4 + \gamma_{42}P^4R^2 + \cdots,$$

where γ_{ij} is a coefficient of the P^iR^j term.

First, we performed the fitting analyses of the $G_{\mathrm{LGD}}(P, R)$ function with various forms of the G_{c} term to the $G_{\mathrm{DFT}}(g, m)$ values (180 points) in the (g, m) subspace. The relationship between (P, R) and (g, m) is expressed in Eqs. 3 and 4. The case of $G_{\mathrm{c}} = \gamma_{22}P^2R^2$ leads to a poor goodness-of-fit with a large chi-square χ^2 of 0.14. The addition of $\gamma_{24}P^2R^4$ does not improve the fit, and χ^2 becomes 0.14 for $G_{\mathrm{c}} = \gamma_{22}P^2R^2 + \gamma_{24}P^2R^4$. The choice of $\gamma_{22}P^2R^2 + \gamma_{42}P^4R^2$ produces a satisfactory result, with a χ^2 of 0.03 (see Fig. 6a). Therefore, we adopt the coupling term of $G_{\mathrm{c}} = \gamma_{22}P^2R^2 + \gamma_{42}P^4R^2$.

Here, we discuss the reason that $\gamma_{42}P^4R^2$ is required to reproduce the G_{DFT} potential. There are several requirements for the G_{LGD} function. In the region of $P \geq 0$ and $R \geq 0$, two local minima, corresponding to the T' and T phases, exist. The T' phase features a small P associated with a certain degree of R, and the T phase exhibits a large P without octahedral rotation (R = 0). The presence of the T' phase entails a saddle point at P = 0 and $R \sim R_0$. Moreover, the T' and T phases are connected through a first-order phase transition with a relatively low energy barrier; therefore, a distinct saddle point exists in the energy valley between these two phases. Supplementary Figure S4a shows a typical G_{LGD} map ($P \geq 0$ and $R \geq 0$) fulfilling the above requirements, which involves a local maximum (denoted by C), two

local minima (T and T') and two saddle points (S_1 and S_2). In this map, the polarization P (horizontal axis) is normalized by P_0 of the point T, and the octahedral rotation angle R (vertical axis) is normalized by R_0 of the point S_1. The stationary points (local extrema and saddle points) satisfy the following conditions:

$$\frac{\partial G}{\partial P} = 0 \Leftrightarrow P = 0 \text{ or } F_1(u,v) = \alpha_2 + \alpha_4 u + 2\gamma_{22} v + 4\gamma_{42} uv = 0,$$

$$\frac{\partial G}{\partial R} = 0 \Leftrightarrow R = 0 \text{ or } F_2(u,v) = \beta_2 + \beta_4 v + 2\gamma_{22} u + 2\gamma_{42} u^2 = 0,$$

where $u = P^2$ and $v = R^2$. If $\gamma_{42} = 0$, the equations of $F_1(u,v) = 0$ and $F_2(u,v) = 0$ represent two straight lines in the (u,v) space and provide at most one common solution in the region of $u > 0$ and $v > 0$ (i.e., $P \neq 0$ and $R \neq 0$). This G_{LGD} function without $\gamma_{42}P^4R^2$ does not represent the G_{DFT} potential, which has stationary points at T' and S_2 in $P > 0$ and $R > 0$ (see Supplementary Fig. S4a). Given that $\gamma_{42} > 0$, two common solutions for $F_1(u,v) = 0$ and $F_2(u,v) = 0$ exist, as depicted in Supplementary Fig. S4b. In this case, the G_{LGD} function contains five stationary points: three are on-axis (S_1, T and C), and two are off-axis (T' and S_2), leading to a good fit to the G_{DFT} values. We optimized the free parameters by fitting the G_{LGD} function to the G_{DFT} potential using the generalized reduced gradient method. The obtained parameters are listed in Table S1. According to the results of the T' and T phases obtained by the DFT calculations (Fig. 4), we here fix the R value of the T' phase (R_a) at 6.54°, and the P value of the T phase (P_b) at 95.1 μC/cm^2.

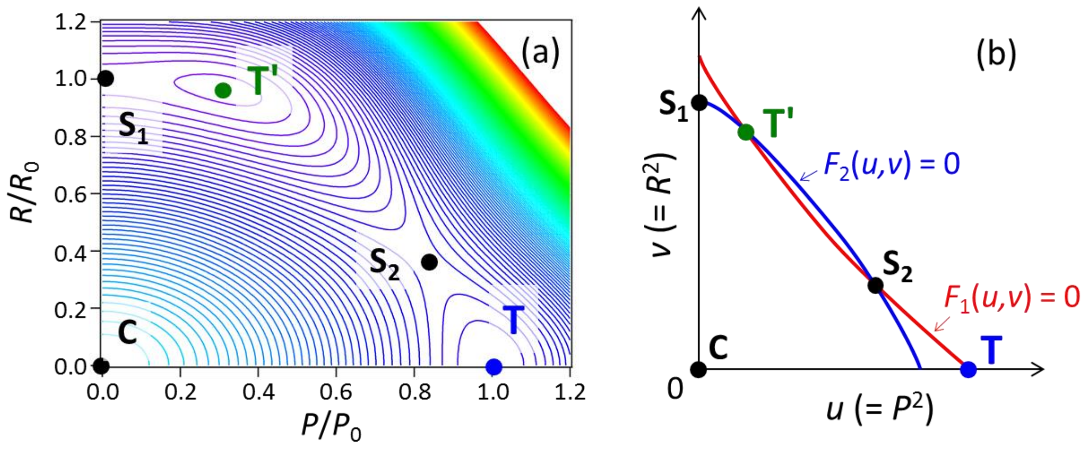

Supplementary Figure S4. (**a**) Two dimensional G_{LGD} map as functions of polarization P and octahedral rotation R obtained by using the function including the coupling term of $G_c = \gamma_{22}P^2R^2 + \gamma_{42}P^4R^2$. (**b**) Stationary points in the (u, v) space when $\gamma_{42} > 0$. Closed circles

indicate the stationary points.

Supplementary Table S1. Parameters of the G_{LGD} function obtained by the fitting analysis.

	Value	unit
G_0	-34.5	eV
α_2	-1.03×10^{-4}	eVcm^4/(μC)^2
α_4	1.15×10^{-8}	eVcm^8/(μC)^4
β_2	-2.21×10^{-2}	eV/deg^2
β_4	4.43×10^{-4}	eV/deg^4
γ_{22}	8.45×10^{-7}	eVcm^4/(μC)^{2}deg^2
γ_{42}	8.41×10^{-11}	eVcm^8/(μC)^{4}deg^2
P_a	36.3	μC/cm^2
R_a	6.54 (fix)	deg
P_b	95.1 (fix)	μC/cm^2

5. LGD calculations for the switching path of the T'-T phase transition

Supplementary Figure S5 displays the switching path of the phase transitions between the T' and T phases induced by electric fields obtained by the LGD theory, taking electric fields into account. We evaluate the variation of the T' and T phases under a sequence of E values by solving a system of equations: $\partial G_{\mathrm{LGD}}/\partial P = E$ and $\partial G_{\mathrm{LGD}}/\partial R = 0$. The relationship between (P, R) and (g, m) is described in Eqs. 3 and 4. The points satisfying the equations are plotted in the (g, m) subspace on the basal plane. On the G_{LGD} surface at E = 0, the energy curves corresponding to the obtained (g, m) points are traced to discuss the implications of the switching path. The T' phase has a flat free-energy surface compared with that of the T phase. With increasing E, the T' phase moves in the (g, m) subspace along the energy valley and subsequently changes to the T phase. The energy slope along the energy valley is much steeper in the T phase than in the T' phase. The movement of the T phase is relatively restricted because of its deep energy potential along the polar (g) axis. With decreasing E, the T phase descends the slope and returns to the T' phase.

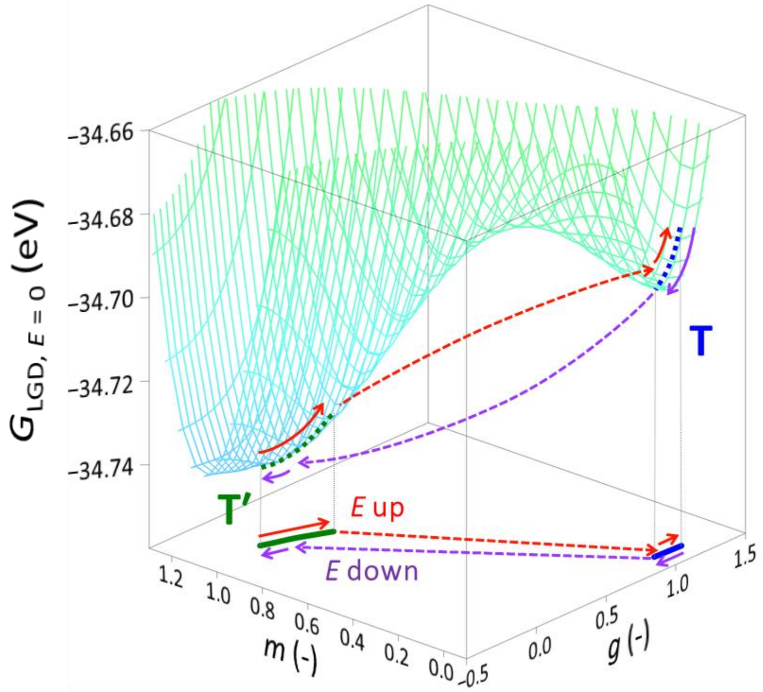

Supplementary Figure S5. Switching path of the phase transitions between the T' and T phases induced by electric fields obtained by the LDG theory. In the (g, m) subspace on the basal plane,

the local minima calculated by taking into account electric fields are plotted. On the G_{LGD} surface at $E = 0$, the energy curves corresponding to the obtained (g, m) minima are traced for the sake of argument. Upward and downward solid arrows indicate the switching paths with increasing and decreasing E, respectively. Dashed arrows show the E-induced phase transitions that are defined to occur once the energy barrier between them becomes lower than 12 meV.

6. Construction of the model structures for the DFT calculations

We considered the BNT structures with different symmetry; $R3c$ [rhombohedral (R), ferroelectric], $P4mm$ [tetragonal (T), ferroelectric], $P4bm$ [tetragonal (T'), ferrielectric], and $Pm\bar{3}m$ [cubic (C), paraelectric]. The rock-salt-like ordering of Bi and Na on the A site lowers the symmetry from $R3c$ to $R3$ for the R phase, $P4mm$ to $I4mm$ for the T phase, $P4bm$ to $P4_2nm$ for the T' phase and $Pm\bar{3}m$ to $Fm\bar{3}m$ for the C phase. We carried out the structural optimizations of the cell shape and fractional coordinates at a fixed cell volume (V_{cell}) under the constraint of the lowered symmetry. The calculated total energy (U_{total}) per perovskite unit as a function of V_{cell} is represented in Supplementary Fig. S6. The solid lines show the fitting curves obtained from the Birch equation of state. The U_{total} values of the R, T, and C phases are in good agreement with the fitting results in all of the V_{cell} range. As for the T' phase, the U_{total} variation is well traced by the fitting curve at the small V_{cell} side. We note that the U_{total} values of the T' phase are apart from the fitting curve in the V_{cell} range of over 58×10^{-3} nm^3 and are close to those of the T phase. At the large V_{cell} side, the T' phase after the optimizations exhibits no significant difference in its structure from the T phase and has no octahedral tilt, leading to almost the same U_{total} for the T' and T phases. Because the T' phase has lower symmetry than the T phase, the crystal structure of the T phase is, in principle, constructed in the T' phase. The constraint of the T'-phase symmetry cannot, therefore, prohibit the structural change to the T phase at the large V_{cell} side. The extrapolated curve obtained from the Birch equation of state can be regarded as the V_{cell} dependence of U_{total} for the T' phase at the large V_{cell} side.

In order to characterize the T'-T phase transition, the crystal structures of the T and T' phases are simplified by the following procedures. Supplementary Tables S2 and S3 list the fractional coordinates of the T and T' phases, respectively, which were used for the calculations of the U_{total}-V_{cell} relationships (Supplementary Fig. S6). These unit cells can be transformed to the tetragonal supercell (T_{super}) [see Table 1] by imposing an additional constraint of $x_{Oa} = 0$ for the T phase and $x_{Oa} = (x_{Oa1}+x_{Oa2})/2$ for the T' phase. These constraints are adopted to restrict a shrinkage of the BiO_{12} dodecahedra on the a-a plane arising from the A-site ordering in the T_{super} cell. This restriction allows us to obtain the T_{super} cell containing only two kinds of atomic displacements: the polar displacement along the c axis and the TiO_6 octahedral tilting around the

c axis.

Here, we introduce a hydrostatic pressure (p_h) into the DFT calculations in order to investigate the phase transition between the T' and T phase. The application of p_h provides the energetically competing T' and T phases while, in reality, the partial occupation of Ba on the A-site in the BNT crystals leads to the *P*4*bm* phase that is slightly stabilized with respect to the *P*4*mm* phase at around room temperature. The curves of total free energy (G_{DFT}) at various p_h values (E = 0), which is defined as $G_{\mathrm{total}} = U_{\mathrm{total}} + p_{\mathrm{h}} V_{\mathrm{cell}}$, are shown in Supplementary Fig. S6 for the T and T' phases in the T_{super} cell. The T' phase features a smaller V_{cell} due to a structural degree of freedom that accommodates the TiO_6 tilting, and its G_{total} is lowered at the small V_{cell} side. At p_h = 0.0 GPa (Supplementary Fig. S6a), the minimum in the G_{total} curve of the T phase is much smaller than that of the T' phase. With increasing p_h, the difference in these two minima becomes small and then almost zero at p_h = 3.2 GPa (Supplementary Fig. S6b). The application of p_h = 6.0 GPa (Supplementary Fig. S6c) yields a reversal of the energy relation and the T' phase exhibits a low G_{total} minimum compared to the T phase. Here, we adopt p_h = 6.0 GPa to investigate the T'-T phase transition under an application of *E*. We confirm that a similar relationship between the T' and T phases appears in the calculations using not only the PBEsol functional but also other exchange-correlation potentials (LDA, PBE, and AM05). We thus conclude that the T_{super} cell prefers to form the T' phase at a higher p_h without depending on the over/under binding tendency of the DFT functionals.

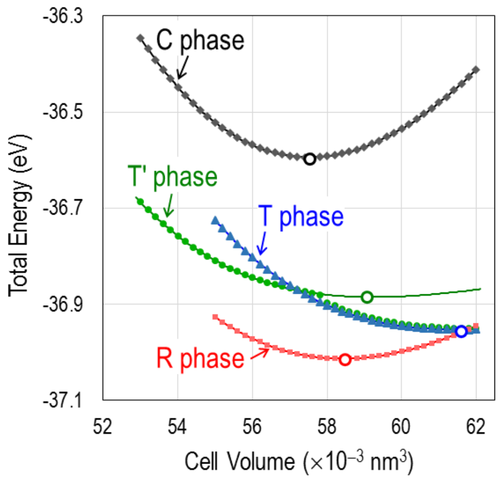

Supplementary Figure S6. Calculated total energies (U_{total}) of BNT with different phases as a function of cell volume (V_{cell}). Solid lines depict the fitting curves obtained from the Birch

equation of state and open circles the minimum points of the fitting curves.

Supplementary Table S2. Sites and coordinates of the constituent atoms in the T-phase unit cell (space group: $I4mm$).

	site	x	y	z
Na	2a	0	0	$1/4 + z_{Na}$
Bi	2a	0	0	$3/4 + z_{Bi}$
Ti	4b	0	1/2	z_{Ti}
O_c	4b	0	1/2	$1/4 + z_{Oc}$
O_a	8c	$1/4 + x_{Oa}$	$1/4 + x_{Oa}$	0

Supplementary Table S3. Sites and coordinates of the constituent atoms in the T'-phase unit cell (space group: $P4_2nm$).

	site	x	y	z
Na	2a	0	0	$1/4 + z_{Na}$
Bi	2a	0	0	$3/4 + z_{Bi}$
Ti	4b	0	1/2	z_{Ti}
O_c	4b	0	1/2	$1/4 + z_{Oc}$
O_{a1}	4c	$1/4 + x_{Oa}$	$1/4 + x_{Oa}$	0
O_{a2}	4c	$3/4 + x_{Oa}$	$1/4 - x_{Oa}$	0

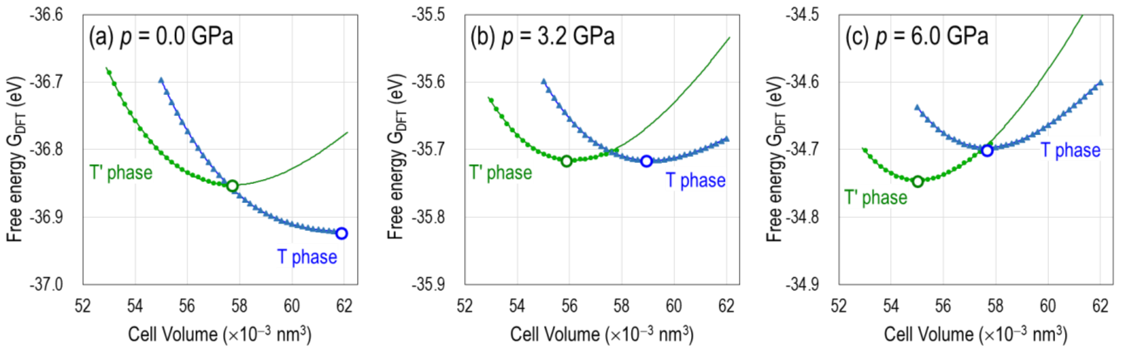

Supplementary Figure S7. Total free energy (G_{DFT}) of the T and T' phases calculated by using the T_{super} cell as a function of cell volume (V_{cell}) at a different hydrostatic pressure (p_h) of (**a**) 0.0 Gpa, (**b**) 3.2 GPa and (**c**) 6.0 GPa.

Supplementary References:

1. Ma, C., Guo, H., Beckman, S. P. & Tan, X. Creation and Destruction of Morphotropic Phase Boundaries through Electrical Poling: A Case Study of Lead-Free $(Bi_{1/2}Na_{1/2})TiO_3$-$BaTiO_3$ Piezoelectrics. *Phys. Rev. Lett.* **109,** 107602 (2012).
2. Ma, C., Guo, H. & Tan, X. A New Phase Boundary in $(Bi_{1/2}Na_{1/2})TiO_3$–$BaTiO_3$ Revealed via a Novel Method of Electron Diffraction Analysis. *Adv. Funct. Mater.* **23,** 5261–5266 (2013).
3. Gorfman, S. & Thomas, P. A. Evidence for a non-rhombohedral average structure in the lead-free piezoelectric material $Na_{0.5}Bi_{0.5}TiO_3$. *J. Appl. Crystallogr.* **43,** 1409–1414 (2010).
4. Aksel, E. *et al.* Monoclinic crystal structure of polycrystalline $Na_{0.5}Bi_{0.5}TiO_3$. *Appl. Phys. Lett.* **98,** 152901 (2011).
5. Jones, G. O. & Thomas, P. A. Investigation of the structure and phase transitions in the novel A-site substituted distorted perovskite compound $Na_{0.5}Bi_{0.5}TiO_3$. *Acta Crystallogr. Sect. B Struct. Sci.* **58,** 168–178 (2002).
6. Glazer, A. M. The classification of tilted octahedra in perovskites. *Acta Crystallogr. Sect. B Struct. Crystallogr. Cryst. Chem.* **28,** 3384–3392 (1972).
7. Ogino, M. *et al.* Polarization Rotation and Monoclinic Distortion in Ferroelectric $(Bi_{0.5}Na_{0.5})TiO_3$–$BaTiO_3$ Single Crystals under Electric Fields. *Crystals* **4,** 273–295 (2014).
8. Kitanaka, Y. *et al.* Crystal Structural Analyses of Ferrielectric Tetragonal $(Bi_{1/2}Na_{1/2})TiO_3$–7%$BaTiO_3$ Powders and Single Crystals. *Jpn. J. Appl. Phys.* **52,** 09KD01 (2013).
9. Onozuka, H., Kitanaka, Y., Noguchi, Y. & Miyayama, M. Crystal Growth and Characterization of $(Bi_{0.5}Na_{0.5})TiO_3$–$BaTiO_3$ Single Crystals Obtained by a Top-Seeded Solution Growth Method under High-Pressure Oxygen Atmosphere. *Jpn. J. Appl. Phys.* **50,** 09NE07 (2011).
10. Rabe, K. M. *Antiferroelectricity in Oxides: A Reexamination* in Functional Metal Oxides, 221–244 (Wiley-VCH Verlag GmbH & Co. KGaA, 2013)
11. Campbell, B. J., Stokes, H. T., Tanner, D. E. & Hatch, D. M. ISODISPLACE: a web-based tool for exploring structural distortions. *J. Appl. Crystallogr.* **39,** 607–614 (2006).
12. Orobengoa, D., Capillas, C., Aroyo, M. I. & Perez-Mato, J. M. AMPLIMODES : symmetry-mode analysis on the Bilbao Crystallographic Server. *J. Appl. Crystallogr.* **42,** 820–833 (2009).
